# Classification of Different Therapeutic Responses of Major Depressive Disorder with Multivariate Pattern Analysis Method Based on Structural MR Scans

**DOI:** 10.1371/journal.pone.0040968

**Published:** 2012-07-17

**Authors:** Feng Liu, Wenbin Guo, Dengmiao Yu, Qing Gao, Keming Gao, Zhimin Xue, Handan Du, Jianwei Zhang, Changlian Tan, Zhening Liu, Jingping Zhao, Huafu Chen

**Affiliations:** 1 Key Laboratory for NeuroInformation of Ministry of Education, School of Life Science and Technology, University of Electronic Science and Technology of China, Chengdu, Sichuan, China; 2 Mental Health Institute, the Second Xiangya Hospital, Central South University, Changsha, Hunan, China; 3 Mental Health Center, the First Affiliated Hospital, Guangxi Medical University, Nanning, Guangxi, China; 4 School of Mathematical Sciences, University of Electronic Science and Technology of China, Chengdu, China; 5 The Mood and Anxiety Clinic in the Mood Disorders Program of the Department of Psychiatry at Case Western Reserve University School of Medicine/University Hospitals Case Medical Center, Cleveland, Ohio, United States of America; 6 Department of Radiology, the Second Xiangya Hospital, Central South University, Changsha, Hunan, China; Institution of Automation, CAS, China

## Abstract

**Background:**

Previous studies have found numerous brain changes in patients with major depressive disorder (MDD), but no neurological biomarker has been developed to diagnose depression or to predict responses to antidepressants. In the present study, we used multivariate pattern analysis (MVPA) to classify MDD patients with different therapeutic responses and healthy controls and to explore the diagnostic and prognostic value of structural neuroimaging data of MDD.

**Methodology/Principal Findings:**

Eighteen patients with treatment-resistant depression (TRD), 17 patients with treatment-sensitive depression (TSD) and 17 matched healthy controls were scanned using structural MRI. Voxel-based morphometry, together with a modified MVPA technique which combined searchlight algorithm and principal component analysis (PCA), was used to classify the subjects with TRD, those with TSD and healthy controls. The results revealed that both gray matter (GM) and white matter (WM) of frontal, temporal, parietal and occipital brain regions as well as cerebellum structures had a high classification power in patients with MDD. The accuracy of the GM and WM that correctly discriminated TRD patients from TSD patients was both 82.9%. Meanwhile, the accuracy of the GM that correctly discriminated TRD or TSD patients from healthy controls were 85.7% and 82.4%, respectively; and the WM that correctly discriminated TRD or TSD patients from healthy controls were 85.7% and 91.2%, respectively.

**Conclusions/Significance:**

These results suggest that structural MRI with MVPA might be a useful and reliable method to study the neuroanatomical changes to differentiate patients with MDD from healthy controls and patients with TRD from those with TSD. This method might also be useful to study potential brain regions associated with treatment response in patients with MDD.

## Introduction

Major depressive disorder (MDD) is characterized by persistent and overwhelming feelings of guilt, sadness, anhedonia, worthlessness, and hopelessness. It is the most common affective disorders and one of the most common psychiatric disorders. It has been estimated that the global disease burden from MDD will be second only to heart disease by the year 2020 [1]. Among the patients with MDD, about one-third of them do not respond to the standard antidepressant treatments. Patients who do not respond to a series of standard antidepressant treatments are commonly defined as having treatment-resistant depression (TRD); meanwhile those who respond to antidepressant treatments defined as having treatment-sensitive depression (TSD) [2]. At present, the diagnosis of MDD is mainly based on clinical signs and symptoms, and treatment protocols are established based on clinical empirical evidence [3], [4]. The etiology and pathogenesis of different phenotypes of MDD remain unknown. Undoubtedly, exploration of neurological biomarker for diagnosis and treatment of MDD has the potential to improve the treatment outcome of patients with MDD.

Over the past several decades, researchers have studied structural and morphometric changes in patients with MDD. Abnormalities in the hippocampus [5], obitofrontal cortex [6], anterior and posterior cingulate cortex (PCC) [7], and cerebellum [8] in patients with MDD have been reported, but the findings are inconsistent. In addition, a recent meta-analysis of voxel-based imaging study find that gray matter (GM) volume reductions in anterior cingulate cortex (ACC), dorsolateral and dorsomedial prefrontal cortex [9]. However, these studies are based on univariate voxel-based analysis. Voxel-based methods only provide limited information because they need more correction for multiple comparisons. Studies using voxel-based analysis require a large sample size to attain sufficient statistical power [10]. Therefore, it is quite possible that the inconsistent findings from previous studies are at least partly due to the use of voxel-based analysis. Moreover, the univariate voxel-based methods do not provide a mechanism for making MDD predictions at the individual level [11].

To overcome the limitations of the univariate voxel-based analysis, the multivariate pattern analysis (MVPA), a data-driven technique, has been used to differentiate psychiatric patients from healthy controls through structural or functional brain images [12], [13], [14]. In contrast to treating each voxel independently in voxel-based analysis, the MVPA assesses contributions of multiple voxels simultaneously to best classify a group so that it may be particularly useful to detect subtle and spatially distributed discriminative patterns in the brain [10], [15]. Specifically, the MVPA not only can find potential neuroimaging-based biomarkers to differentiate patients from healthy controls at the individual level, but also potentially detect spatially distributed information to further highlight the neural mechanisms underlying the pathophysiology of major depression [16].

To date, few studies have used the MVPA technique in the analysis of structural MRI data in depression with only two studies including the analysis for different therapeutic responses [3], [17]. In the present study, we applied a modified MVPA method that combined searchlight algorithm and principal component analysis (PCA) to classify subjects with TRD and those with TSD from matched healthy subjects, and to investigate the diagnostic and prognostic value of structural MRI data of MDD patients. We hypothesized that structural MRI with the MVPA analysis had discriminative effect on diagnosis and treatment response in patients with MDD.

## Materials and Methods

### Subjects

The present study was approved by the Ethics Committee of the Second Xiangya Hospital of the Central South University, China. Written informed consents were obtained from all subjects before any study procedure was initiated. Eighteen right-handed TRD patients were recruited from the Mental Health Institute of the Second Xiangya Hospital of the Central South University, China. The patients were partially from one of our previous studies [18]. MDD was diagnosed by two research psychiatrists (Dr Zhao J and Dr Liu Z) using the Structured Clinical Interview according to the DSM-IV criteria [19]. Exclusion criteria included age younger than 18 years or older than 50 years, any history of major physical illness, cardiovascular disease, bipolar disorder, neurological illness, or a lifetime history of alcohol or drug use. Severity of depression was assessed with the 17-item Hamilton Rating Scale for Depression (HRSD) [20]. All patients had taken at least two classes of antidepressants before being enrolled in the study. The TRD was defined as a poor response to at least two adequate trials (adequate dosages, duration, and compliance) with different classes of antidepressants [21], [22]. The poor response was defined as less than 50% reduction in the HRSD total score after treatment at a minimum dose of 150 mg/day of imipramine or the equivalents (dose converted using a conversion table) for 6 weeks [23].

Twenty-four right-handed TSD patients partially from a different previous study [24] were included for this analysis. All patients were treatment-naive and at their first episode of MDD. Exclusion criteria were similar to those of TRD patients. An additional exclusion criterion for these patients was that the current illness duration was more than six months. The severity of depression was also quantified with the 17-item Hamilton Rating Scale for Depression (HRSD). Shortly after baseline MRI scanning was completed, all patients were prescribed an antidepressant at a minimum dose of 150 mg/day of imipramine equivalents (dose converted using a conversion table) for 6 weeks by the same psychiatrists (Dr Zhao J and Dr Liu Z) [25]. For those who had a reduction in HRSD total score of more than 50% after the antidepressant treatment, they were defined as having TSD. This practice was consistent with previous studies [17], [21], [22], [26].

Seventeen right-handed healthy controls came from the same study as those with TRD [18]. They were recruited from the local community through advertisements. They were also screened by the same psychiatrists (Dr Zhao J and Dr Liu Z) with the Structured Clinical Interview for DSM-IV, non-patient edition. None of them had serious medical or neuropsychiatric illness. There was no major psychiatric or neurological illness in their first-degree relatives.

### MRI Data Acquisition

A 1.5T GE scanner (General Electric, Fairfield, Connecticut, USA) with a volumetric 3D Spoiled Gradient Recall (SPGR) sequence was used to scan all participants. The acquisition parameters were: repetition time/echo time (TR/TE) = 12.1/4.2 ms, flip angle = 15°, field of view = 240 mm×240 mm, image matrix = 512×512×172, voxel size = 0.51×0.51×0.9 mm^3^.

### Image Processing

All images were visually inspected for artifacts or structural abnormalities before voxel-based morphometry (VBM) analysis was applied to the structural MRI images by using SPM8 (Wellcome Trust Centre for Neuroimaging, Institute of Neurology, UCL, London, UK; http://www.fil.ion.uncl.ac.uk/spm). The detailed steps of VBM analysis were as follows. First, all structural images were manually set the origin to the anterior commissure. Second, all images were segmented into GM, white matter (WM), and cerebrospinal fluid (CSF) and imported into a rigidly aligned space [27]. Third, the segmented images were iteratively registered by the Diffeomorphic Anatomical Registration Through Exponentiated Lie algebra (DARTEL) toolbox [28]. This procedure generated a template for a group of individuals. Fourth, the resulting images were spatially normalized into the MNI space using an affine spatial normalization. An additional processing step consisted of multiplying each spatially normalized image by its relative volume before and after normalization with the purpose of preserving the total amount of each tissue. Finally, the images were smoothed with an 8 mm full width at half maximum (FWHM) isotropic Gaussian kernel.

### Multivariate Pattern Analysis

**Figure 1:**
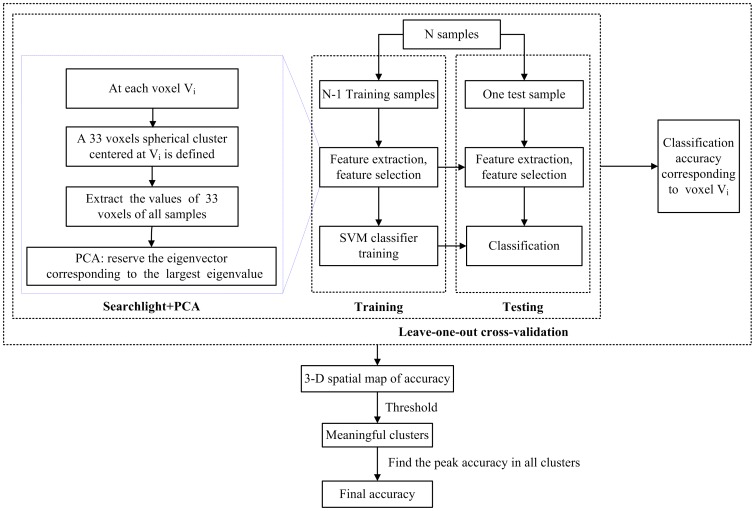
Flow chart of the proposed MVPA method.

In this study, we used a modified MVPA technique that combined searchlight algorithm and PCA. The searchlight technique was proposed by Kriegeskorte et al. [29] and has been widely used in neuroimaging studies because of its superior ability to extract features as the input of pattern analysis [30], [31]. Uddin and colleagues recently used this technique to discriminate children and adolescents with autism from healthy subjects [31]. The procedure of modified MVPA method was as follows. The inputs into MVPA were the smoothed tissue maps (i.e. GM or WM) that were generated after image processing procedure, and then all maps were divided into training set and testing set. At each voxel V_i_, a 33 voxels spherical cluster centered at V_i_ was defined (according to the searchlight algorithm with optimal or near-optimal detection performance [29] ). The values of the voxels in the spherical cluster were extracted, and then data matrices W_N1×V_ and W_N2×V_ were acquired for training set and testing set, respectively (N1 and N2 were the number of subjects in the two sets, and V was the number of the voxels in the cluster). Subsequently, PCA was applied as a dimension reduction way to reduce the data matrix to its eigenvectors in the training set and testing set, respectively. Only the eigenvector E_S×1_ having the largest eigenvalue was reserved as the final classification feature (see Figure S1 for detailed analysis). Finally, a linear support vector machine (SVM) classifier was performed using LIBSVM software (Software available at http://www.csie.ntu.edu.tw/~cjlin/libsvm). To estimate the performance of our classifier, a leave-one-out cross-validation (LOO-CV) test was used to assess the overall accuracy of the classifier in the present study. Briefly, if there are *N* samples in total, in each LOO-CV experiment, the *N−1* samples are viewed as the training set, and the omitted one is used as a test subject to computing the classification error. LOO-CV accuracy for voxel V_i_ was yielded by averaging all accuracies obtained at each tested subject. The resulting three-dimensional spatial map of LOO-CV accuracy at each voxel was used to detect brain regions that exhibited differences between the two participant groups. The flow chart of aforementioned method was shown in Figure 1. Compared with a previous similar study [30], a more rigorous threshold was used in our study. A meaningful cluster of these three-dimensional spatial accuracy maps was considered as accuracies higher than 70% (higher than the chance level of 50%) and contiguous voxels with at least 50 voxels. Moreover, like previous studies [30], [31], the overall accuracy in our study was the peak accuracy of all clusters identified.

To evaluate the statistical significance of the accuracies in each cluster, the permutation test was used [32]. In permutation test, the class labels of the training data were randomly permuted before training. Cross-validation was performed on the permuted training set, and the permutation was repeated 1000 times. The accuracy values were obtained from all permutations. The proportion of the accuracy values that were equal to or greater than the value generated by the non-permutated data was calculated. If less than 5% (*p*<.05) of the values from all permutations exceeded the actual value, the accuracy was considered statistically significant.

### Voxel-based Analysis

To investigate the alterations of GM/WM volumes in the identified brain regions of patients with TRD or TSD, between-group comparisons of GM/WM volumes were performed with two-sample *t* tests on smoothed images within a mask. This mask was created by the identified brain regions using aforementioned MVPA method between TRD and TSD patients. Outcomes were assessed at two different statistical thresholds: (1) *p*<.05, family error rate (FWE) corrected; (2) *p*<.001, uncorrected.

### Correlation Analysis

To explore whether the identified important GM and WM regions between TRD and TSD patients were correlated with the severity of depressive symptoms, voxel-based correlation analyses were applied to all voxels in the abnormal areas of the GM and WM and the HRSD total scores. Using the AlphaSim program in the REST software (http://sourceforge.net/projects/resting-fmri), the resulting statistical map was corrected for multiple comparisons to a significant level of *p*<.05 (combined height threshold *p*<.02 and a minimum cluster size of 10 voxels).

### Comparison with Other MVPA Methods

To better understand the performance of our MVPA technique, we compared the findings of the MVPA method in the present study with those of other MVPA methods to the same structural data. Other MVPA methods included recursive feature elimination (RFE), locally linear embedding (LLE) and C-means, and LLE+linear SVM. The RFE, a feature ranking method based on SVM, has been successfully applied in other neuroimaging studies [10], [33]. Similarly, the LLE and C-means classifier has also been successfully used to distinguish schizophrenia patients from healthy controls [34].

## Results

### Demographics and Clinical Characteristics of the Participants

The demographic and clinical data are presented in Table 1. Gender, age and the years of education did not differ significantly among the three groups. There was no significant difference in baseline HRSD score between TRD and TSD. The course of disease was significantly greater in the TRD group compared to TSD. Eighteen patients with TRD, 17 patients with TSD and 17 healthy subjects were included, but seven patients with TSD were excluded due to not responsive to treatment.

**Table 1:** Demographics and clinical characteristic of patients with MDD and healthy controls.

Characteristics	TRD	TSD	HC	*P* Value
Gender(M/F)	11/7	10/7	10/7	0.987^a^
Age, years	27.39±7.74	26.71±7.73	24.24±4.41	0.368^b^
Education, years	13.56±3.60	12.35±2.12	13.82±2.38	0.271^b^
Course, months	35.5±49.89	2.59±1.33	–	0.010^c^
HRSD	23.89±3.69	25.58±6.32	2.58±1.54	<0.001^b^

HRSD, Hamilton Rating Scale for Depression. TRD, treatment-resistant depression; TSD, treatment-sensitive depression; HC, healthy controls; plus-minus values are Mean±SD.

a The *P* value for gender distribution in the three groups was obtained by chi-square test.

b The *P* values were obtained by one-way analysis of variance tests.

c The *P* values were obtained by two sample *t*-test.

### Accuracy of GW and WM in Discriminating TRD from TSD

**Figure 2:**
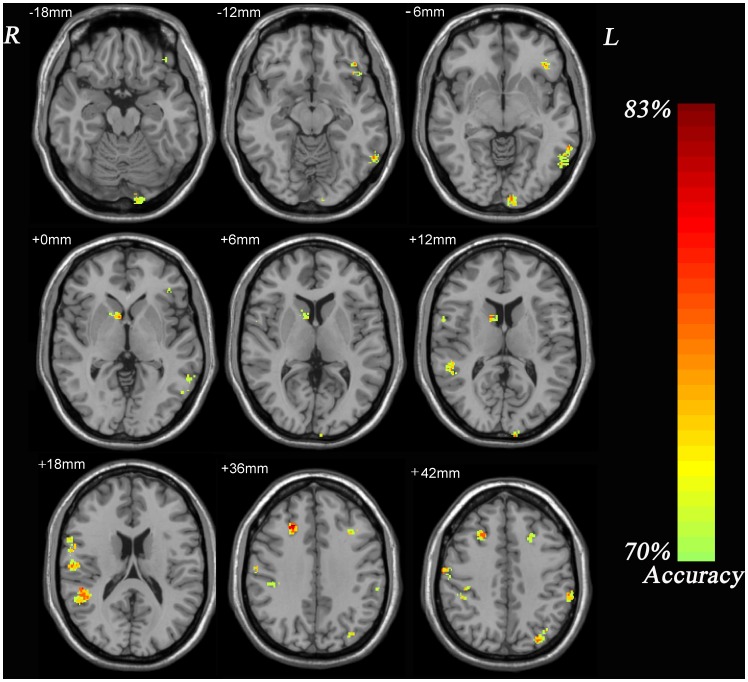
Resulting spatial maps of accuracy for discriminating between TRD patients and TSD patients using gray matter. These clusters were identified by setting the threshold of accuracy higher than 70% and cluster size more than 50 voxels.

**Figure 3:**
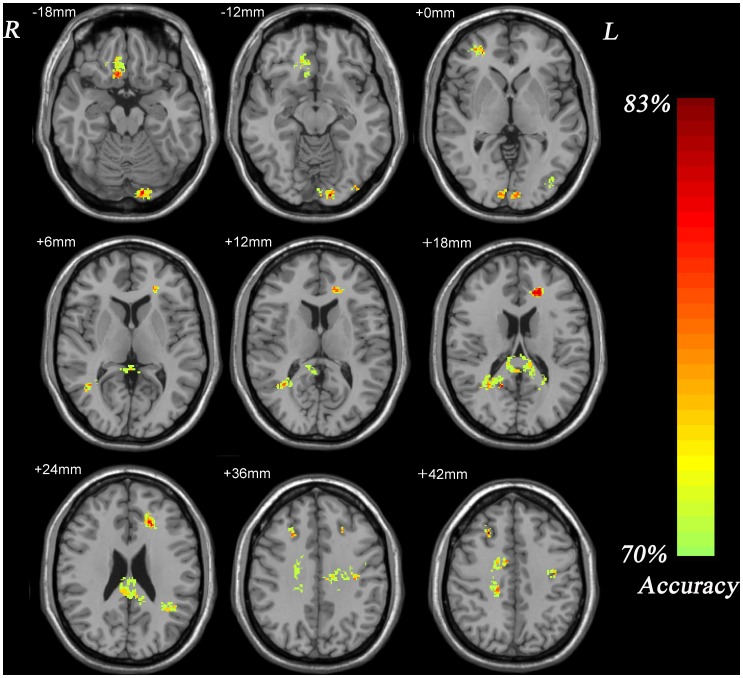
Resulting spatial maps of accuracy for discriminating between TRD patients and TSD patients using white matter. These clusters were identified by setting the threshold of accuracy higher than 70% and cluster size more than 50 voxels.

As shown in Figure 2 and Table 2, several areas of the GM in frontal lobe, parietal lobe, temporal lobe, occipital lobe, and cerebellum had discriminative effect on distinguishing patients with TRD from those with TSD. Similarly, as shown in Figure 3 and Table 3, there were several areas of the WM in each lobe showing significant differences between patients with TRD and those with TSD. As a prognostic marker of treatment response to antidepressants, the accuracy of the GM image and the WM image that correctly discriminated TRD patients from TSD patients was both 82.9% (Table 4).

**Table 2:** Most important gray matter regions discriminating between TRD patients and TSD patients.

Brain regions	BA	Cluster size (voxels)	MNI coordinates (mm)			Peak Accuracy(%)	*P* value
			x	y	z		
**Frontal**							
Left superior frontal gyrus	8	62	−22	22	39	74.2	0.002
Right superior frontal gyrus	8	192	24	24	34	82.9	0.001
Left middle frontal gyrus	9	119	−27	25	28	77.1	0.001
Left inferior frontal gyrus	11/47	233	−39	34	−1	80.0	0.001
Right precentral gyrus	4	161	58	−10	40	80.0	0.001
**Parietal**							
Left precuneus	7/31	146	−30	−78	39	80.0	0.001
Right postcentral gyrus	3	309	60	−18	16	77.1	0.001
Left supramarginal gyrus	40	129	−60	−40	22	80.0	0.001
Right supramarginal gyrus	40	136	46	−37	45	77.1	0.002
Left inferior parietal lobule	39/40	93	−58	−34	42	77.1	0.001
Right inferior parietal lobule	39/40	53	46	−30	33	74.2	0.001
**Occipital**							
Left lingual gyrus	17/18	121	−13	−96	−22	74.2	0.001
Left calcarine fissure	17/18	103	−9	−102	−10	77.1	0.001
Left superior occipital gyrus	18	52	−10	−106	12	77.1	0.001
**Temporal**							
Right superior temporal gyrus	22	427	51	−42	16	82.9	0.001
Left middle temporal gyrus	21	60	−58	−9	−25	82.9	0.001
Left inferior temporal gyrus	20	335	−58	−58	−13	80.0	0.001
**Cerebellum**							
Left cerebellum posterior lobe	–	300	−42	−72	−40	77.1	0.001
Right cerebellum posterior lobe	–	128	7	−61	−63	74.2	0.001
**Subcortical**							0.001
Right caudate nucleus	−	189	7	6	1	80.0	0.001

The *P* values were obtained by permutation test. BA, Broadmann’s area.

**Table 3:** Most important white matter regions discriminating between TRD patients and TSD patients.

Brain regions	BA	Cluster size (voxels)	MNI coordinates (mm)			Peak Accuracy(%)	*P* value
			x	y	z		
**Frontal**							
Right medial frontal gyrus	25	459	12	21	−19	80.0	0.001
Right middle frontal gyrus	8/9	140	25	25	42	82.9	0.001
Right middle frontal gyrus	10	239	25	45	−4	80.0	0.001
Left anterior cingulate gyrus	32	678	−18	33	18	82.9	0.001
Right anterior cingulate gyrus	31/24	416	19	−30	39	77.1	0.001
Left median cingulate gyrus	24	234	−10	−16	39	77.1	0.001
Left precentral gyrus	6	205	−37	−18	37	80.0	0.001
**Parietal**							
Left supramarginal gyrus	40	110	−39	−51	22	77.1	0.001
Left precuneus	7	52	−18	−69	48	80.0	0.001
Left posterior cingulate gyrus	23/31	979	−10	−42	19	77.1	0.001
**Occipital**							
Left lingual gyrus	17/18	487	−15	−91	−21	82.9	0.001
Right lingual gyrus	17/18	110	10	−94	−1	80.0	0.001
Left middle occipital gyrus	19	59	−33	−79	3	80.0	0.001
Left inferior occipital gyrus	18/19	156	−39	−84	−9	80.0	0.001
**Temporal**							
Right middle temporal gyrus	21/22	457	33	−54	10	80.0	0.001

The *P* values were obtained by permutation test. BA, Broadmann’s area.

**Table 4:** Comparison of discriminative performance of different MVPA methods on TRD versus TSD and TRD or TSD versus controls.

Classification feature	Feature selection	Classifier type	Leave-one-out cross-validation		
			TRD vs. TSD	TRD vs. HC	TSD vs. HC
Gray matter	Searchlight+PCA	Linear SVM	82.9%	85.7%	82.4%
White matter	Searchlight+PCA	Linear SVM	82.9%	85.7%	91.2%
Gray matter	RFE	Linear SVM	77.1%	77.1%	70.6%
White matter	RFE	Linear SVM	82.9%	85.7%	76.5%
Gray matter	LLE	C-Means	77.1%	77.1%	76.5%
White matter	LLE	C-Means	65.7%	85.7%	88.2%
Gray matter	LLE	Linear SVM	80.0%	77.1%	82.4%
White matter	LLE	Linear SVM	77.1%	85.7%	88.2%

PCA, Principal component analysis; RFE, recursive feature elimination; LLE, locally linear embedding; TRD, treatment-resistant depression; TSD, treatment-sensitive depression; HC, healthy control.

### Accuracy of GW and WM in Discriminating TRD or TSD from Control

For the diagnostic predictivity, the accuracy of the GM image that correctly discriminated TRD or TSD patients from healthy controls were 85.7% and 82.4%, respectively (Table 4). As shown in Figure S2 and Figure S3, and Table S1 and Table S2, there were several areas showing significant differences between patients with TRD and healthy controls (Figure S2 and Table S1), and patients with TSD and healthy controls (Figure S3 and Table S2). Similarly, the accuracy of the WM image that correctly discriminated TRD or TSD patients from healthy controls were 85.7% and 91.2%, respectively (Table 4). The accuracy maps and areas of the WM distinguishing patients with TRD from the healthy controls were shown in Figure S4 and Table S3 and the accuracy maps and areas of the WM distinguishing patients with TSD from the healthy controls were shown in Figure S5 and Table S4.

### Comparison of Voxel-based Analysis between TRD and TSD

There were no significant volumetric differences in either GM or WM between two groups with an FWE correction rate of *p*<.05. To compare the results obtained with MVPA and traditional univariate analysis of VBM, we further lowered the statistical threshold to a lenient value of *p*<.001 (uncorrected) to detect potential brain regions that might be involved in different therapeutic responses of patients with MDD. However, as shown in Table S5 and Table S6, the VBM detected few differences in small clusters between the two groups.

### Correlations between GM/WM Volume and the Severity of Depression

Correlation analyses were conducted between the GM volumes in the identified areas with MVPA (Table 2) and the HRSD total scores of the pooled patients with MDD. Significantly positive correlations were revealed between the GM volume of the posterior lobe in bilateral cerebellum, the left inferior frontal gyrus, the right superior temporal gyrus, and the left inferior parietal lobule and the HRSD total scores of the pooled patients, while no region showed significantly negative correlation with HRSD score (*p*<.05, AlphaSim corrected; Table S7).

Similarly, correlation analysis of WM volumes in the identified areas with MVPA (Table 3) against the HRSD score showed significantly positive correlations for the right cuenus and significantly negative correlations for the left medial frontal gyrus and left median cingulate gyrus (*p*<.05, AlphaSim corrected; Table S8).

### Comparison with Other MVPA Methods

As we illustrated in the third and fourth row of the Table 4, the RFE approach yielded relatively low classification accuracy. In addition, our method that combined searchlight algorithm and PCA outperformed the LLE+C-means and LLE+linear SVM method (Table 4). These results further validated the potential use of our MVPA method for MDD classification.

## Discussion

In the present study, we have shown that structural MRI with MVPA might be a useful and reliable method to study the neuroanatomical changes to differentiate patients with MDD from healthy controls and patients with TRD from those with TSD. This method might also be useful to study potential brain regions associated with treatment response in patients with MDD. The GM and WM had comparable accuracy to distinguish TRD or TSD from healthy controls and TRD from TSD. Although there were many brain regions showing differences among patients with MDD and healthy controls, the findings support that the neuroanatomical structures of MDD are mainly involved in a series of specific networks that include frontal, temporal, parietal and occipital regions as well as the cerebellum.

The core components of default mode network (DMN), including medial prefrontal cortex (MPFC), PCC/precuneus (PCU), and inferior parietal lobe (IPL) were found to display high diagnostic and prognostic accuracy. Recent studies revealed a key role for the DMN in the pathophysiology of depression. One recent study suggested that depression could be considered as an illness due to the pathological inability of the DMN to adjust self-referential activity in a situationally appropriate manner [35]. In another study, Hamilton et al. demonstrated that patients with MDD had increased levels of DMN dominance which was related to higher levels of maladaptive, depressive rumination and lower levels of adaptive, reflective rumination [36]. In a VBM study, morphologic differences in the MPFC and PCC between patients with late-onset depression and healthy controls were observed [37]. The IPL was thought to play a part in emotional modulation. Compared depressed patients with healthy controls, enhanced activation in IPL during the response to sad words was observed in depressed patients [38]. In geriatric patients with remitted depression, the activation in IPL to sad words was attenuated [39]. The PCU, a part of the parietal lobe, had GM volumetric abnormality in TRD [22], and increased activity after responding to TMS treatments [40]. Results from our present study not only extended the findings from previous studies, but also provided new evidence that the DMN may play an important role in MDD. The structural alterations of the DMN might contribute to the functional abnormalities at the network level. The differences in the structural alterations between patients with TRD and those with TSD suggested that the structural differences might be related to different responses to antidepressant treatments.

The ACC is a key structure in brain networks that are involved in mood regulation [7]. The association between the change in ACC activity and clinical response to antidepressants was reported [41]. In a functional imaging study, Pizzagalli et al. revealed that baseline hyperactivity in the ACC predicted treatment response in acutely depressed patients [42]. Additionally, Chen et al. demonstrated that ACC volumes were positively associated with the speed of antidepressant response [43]. Moreover, in a combined positron emission tomography (PET) and MRI study of mood disorders, Drevets et al. reported that the ACC’s mean gray matter volume was decreased in patients with MDD or bipolar disorder, irrespective of their mood states [44]. In the present study, we found that the WM volume of ACC could discriminate TRD from TSD (Table 3), and TSD from the healthy controls (Table S4) with high accuracy, suggesting that the ACC might be a trait marker for MDD.

The dorsolateral prefrontal cortex (DLPFC, Broadmann’s area 9) is a critical region in the cognitive control networks [45]. This region is involved in the modulation of emotional responses. Dysfunction of this region will result in abnormal physiological and psychological responses to stressful stimuli [46]. Similarly, the role of DLPFC in the pathogenisis of MDD has been investigated with imaging studies [47], [48], [49]. Results from a previous study suggested that the involvement of DLPFC in depression was closely related to the cognitive symptoms of depression [50]. Taken together, our finding of structural alterations in the DLPFC strongly implicate that this region is central to the pathophysiology of MDD.

Since the traditional emphasis on cerebellum function has been the acquisition of motor coordination and motor behavior [51], the relatively high accuracy of the cerebellum on distinguishing patients with TRD from TSD, and patients with MDD from healthy controls was somewhat unexpected. These findings were consistent with previous studies showing that the cerebellum played an important part in the perception of emotional stimuli and emotional control [52], [53]. Anatomically, different regions of the cerebellum like the vermis, fastigial nucleus, and flocculonodular lobe have connections with brainstem reticular nuclei [54] and the limbic system including hippocampus and amygdala, hypothalamus, and periaqueductal gray [55], [56], [57]. Meanwhile, the cerebellum receives projections from the caudal and rostral anterior cingulate via the pons [58]. Hence, these connections may provide an anatomical basis for the cerebellum to play a regulation role in emotion and cognition. So far, several studies have found neuroanatomical differences in the cerebellum of MDD patients. Peng and colleagues documented decreased GM density in the cerebellum in MDD patients compared with healthy subjects [8]. Frodl et al demonstrated that MDD patients had a significantly decreased GM density in cerebellum [59]. A recent meta-analytic study revealed reduced activation of cerebellum posterior lobe to positive emotion in depressed group compared with healthy subjects [60]. In addition, Baillieux et al. observed that patients with cerebellar lesions would lead to a deficit in planning, learning, and attention processes [61]. Thus, these data suggested that structural changes of the cerebellum may result from the emotional and cognitive deficiency that commonly encountered in patients with MDD.

Our results also showed some temporal and occipital regions having high accuracy in discriminating patients with MDD from healthy subjects. The prognostic and diagnostic potential of these regions in patients with MDD are also found in other studies [3], [17]. A previous study concluded that structural abnormalities of the temporal regions might reflect the part of a disturbed neural network of MDD because patients with MDD had a decreased GM density in the temporal lobes [8]. In a single photon emission computed tomography (SPECT) study, occipital lobe perfusion deficits were observed in adolescents and young adults with MDD [62]. Similarly, abnormal spontaneous activity in the bilateral occipital lobes was reported in patient with depression compared with that in healthy controls [63]. These results supported the notion that temporal and occipital regions might also be used to diagnose or predict the treatment response of patients with MDD.

As shown in Table S1, S2, S3, S4, there were more structural abnormal regions between TSD patients and healthy controls than between TRD patients and healthy controls. These findings may be implausible as one might expect that patients with TRD should have more structural alternations relative to healthy controls than patients with TSD do. However, these results are not incredible in the light of previous neuroimaging studies which compared TRD and TSD patients with healthy controls. For example, a VBM study revealed that TSD patients had reduced GM volume in the bilateral medial/superior frontal gyrus and left postcentral gyrus compared with healthy controls, but patients with TRD did dot have significant differences in the GM volume in these regions compared with healthy controls [64]. Similarly, in a resting-state functional connectivity study, Lui and colleagues found that compared to healthy controls, TSD patients had a more distributed decrease in connectivity than TRD patients, especially in the ACC and in the amygdala, hippocampus, and bilateral insula; however, the TRD group had disrupted functional connectivity mainly in prefrontal areas and in bilateral thalamus areas [65]. These findings suggest some alterations in the brain may be unique to TSD patients and others may be unique to TRD patients. Because of this reason, inconsistent findings from pervious studies might be a result of the heterogeneity of studied samples which included both patients with TRD and TSD.

The finding that the traditional VBM approach did not find significant differences in GM/WM volume in any brain region between TRD and TSD patients suggested that VBM method may not be sensitive enough to detect subtle differences between these two conditions. In contrast, the MVPA method used in the present study was able to find significant differences in the GM/WM volume in many brain regions between TRD and TSD patients, which suggested that the MVPA can be used to detect subtle and spatially distributed neuroanatomical differences in different groups of patient with MDD or to study brain regions for predicting therapeutic responses of MDD. In addition, the positive correlations of the GM and WM volume in the identified brain regions in TRD and TSD patients with the severity of depressive symptoms (Table S7 and ) suggested that these regions might be used as quantitative markers for the assessment of depressive symptoms of MDD.

To our knowledge, only two published studies have used structural MRI data to investigate the association between brain structures and therapeutic responses of patients with MDD. The first study employed SVM to GM to examine the predictive potential for clinical response to antidepressant treatment. The accuracy of the whole brain structural neuroanatomy to predict clinical response was 88.9% (*p* = .01). The accuracy as a diagnostic marker for MDD was 67.6% with a sensitivity of 64.9% and a specificity of 70.3% (*p* = .027) [3]. However, the findings from this study may not be generalizable due to a small sample size (*n* = 9 for each group). In another recent study, the diagnostic accuracy of GM and WM was 67.39% and 58.70% for TRD vs. control, 76.09% and 58.70% for TSD vs. control, and 69.57% and 84.65% for TRD vs. TSD respectively [17]. However, the diagnostic accuracy of the WM on discriminating TRD patients from healthy controls is not statistically significant (*p* = .13). In the present study, the accuracy of the GM and WM on discriminating TRD from TSD was both 82.9% (Table 4) and the accuracy of the GM and WM on discriminating TRD or TSD patients from healthy controls were also both over 82.0% (Table 4). These data suggested that the MVPA method might be a more effective and accurate method to study the relationship between brain structures and different therapeutic responses and the differences of brain structures between patients with mood disorder and healthy subjects.

The following limitations should be noted. First, our study was limited by a relatively small sample size. Consequently, our preliminary results must be confirmed with larger sample studies of patients with MDD and healthy control subjects. Second, since all TRD patients were not medication free before participating in this study, therefore, the effect of medication on brain structures could not be excluded; Future studies in drug-free TRD subjects may help to address this issue. Third, the current study was limited by the heterogeneous pharmacological profiles. Patients were treated with one of three different classes of antidepressants. It is quite possible that one patient may exhibit treatment non-response to one antidepressant, but can be treatment-response to another. Therefore, this heterogeneity might limit the generalizability of our findings. For this reason, future studies should use a sequential approach to determine TRD cases. Finally, the TSD group had shorter illness duration than that of the TRD group. However, there is no straightforward way to incorporate illness duration covariates into the MVPA method for the moment. Accordingly, we cannot fully rule out the possibility that our findings were influenced by this variable. In the future, we will devote to improving our method to solve this problem.

In summary, this study used a modified MVPA approach to explore the diagnostic and prognostic potential of structural MRI in patients with MDD. The results demonstrated that the MVPA not only achieved a high accuracy on distinguishing patients with MDD from healthy controls, and TRD from TSD, but also identified brain regions that may be used as biomarkers to diagnose and predict treatment response of MDD. Although we focused on structural MRI here, other modalities such as functional MRI and diffusion MRI will be integrated into MVPA in the future.

## Supporting Information

Figure S1 The accuracy as a function of the number of eigenvector used in classification. TRD, treatment-resistant depression; TSD, treatment-sensitive depression; HC, healthy control; GM, gray matter; WM, white matter.
(TIF)

Figure S2 Resulting spatial maps of accuracy for discriminating between TRD patients and healthy controls using gray matter. These clusters were identified by setting the threshold of accuracy higher than 70% and cluster size more than 50 voxels.
(TIF)

Figure S3 Resulting spatial maps of accuracy for discriminating between TSD patients and healthy controls using gray matter. These clusters were identified by setting the threshold of accuracy higher than 70% and cluster size more than 50 voxels.
(TIF)

Figure S4 Resulting spatial maps of accuracy for discriminating between TRD patients and healthy controls using white matter. These clusters were identified by setting the threshold of accuracy higher than 70% and cluster size more than 50 voxels.
(TIF)

Figure S5 Resulting spatial maps of accuracy for discriminating between TSD patients and healthy controls using white matter. These clusters were identified by setting the threshold of accuracy higher than 70% and cluster size more than 50 voxels.
(TIF)

Table S1 Most important gray matter regions discriminating between TRD patients and healthy controls.
(DOC)

Table S2 Most important gray matter regions discriminating between TSD patients and healthy controls.
(DOC)

Table S3 Most important white matter regions discriminating between TRD patients and healthy controls.
(DOC)

Table S4 Most important white matter regions discriminating between TSD patients and healthy controls.
(DOC)

Table S5 Brain regions showing gray matter volume differences in TRD patients compared with TSD patients.
(DOC)

Table S6 Brain regions showing white matter volume differences between the TRD patients and TSD patients.
(DOC)

Table S7 Correlation between HRSD scores and gray matter volume in TRD and TSD patients.
(DOC)

Table S8 Correlation between HRSD scores and white matter volume in TRD and TSD patients.
(DOC)
