# Supplementary figures and images for: Classification of Different Therapeutic Responses of Major Depressive Disorder with Multivariate Pattern Analysis Method Based on Structural MR Scans
Source: PLoS One. 2012 Jul 17;7(7):e40968. doi: 10.1371/journal.pone.0040968 (PMC3398877; doi:10.1371/journal.pone.0040968)

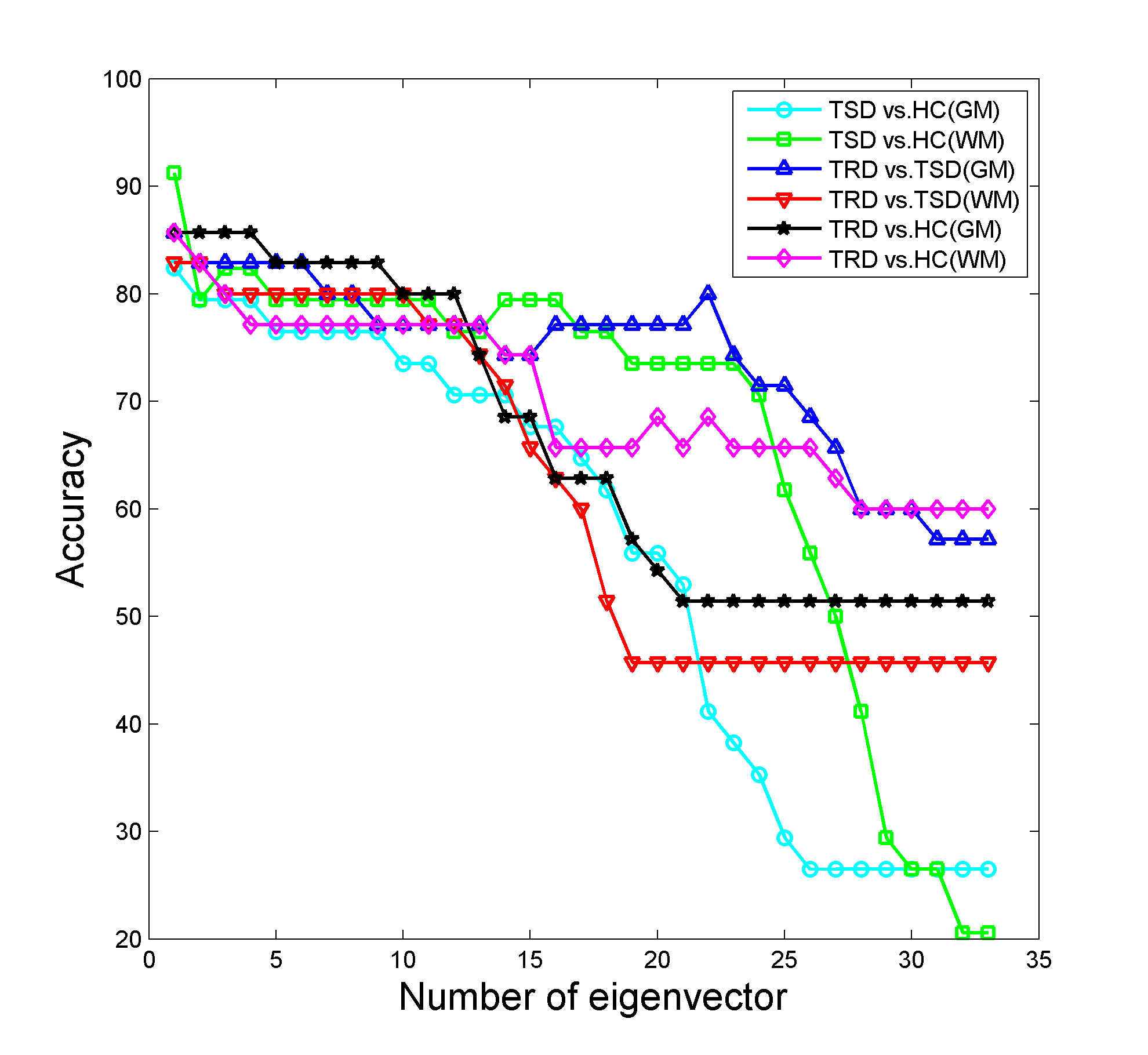

Supplement: Figure S1 — The accuracy as a function of the number of eigenvector used in classification. TRD, treatment-resistant depression; TSD, treatment-sensitive depression; HC, healthy control; GM, gray matter; WM, white matter. (TIF) [file pone.0040968.s001.tif]

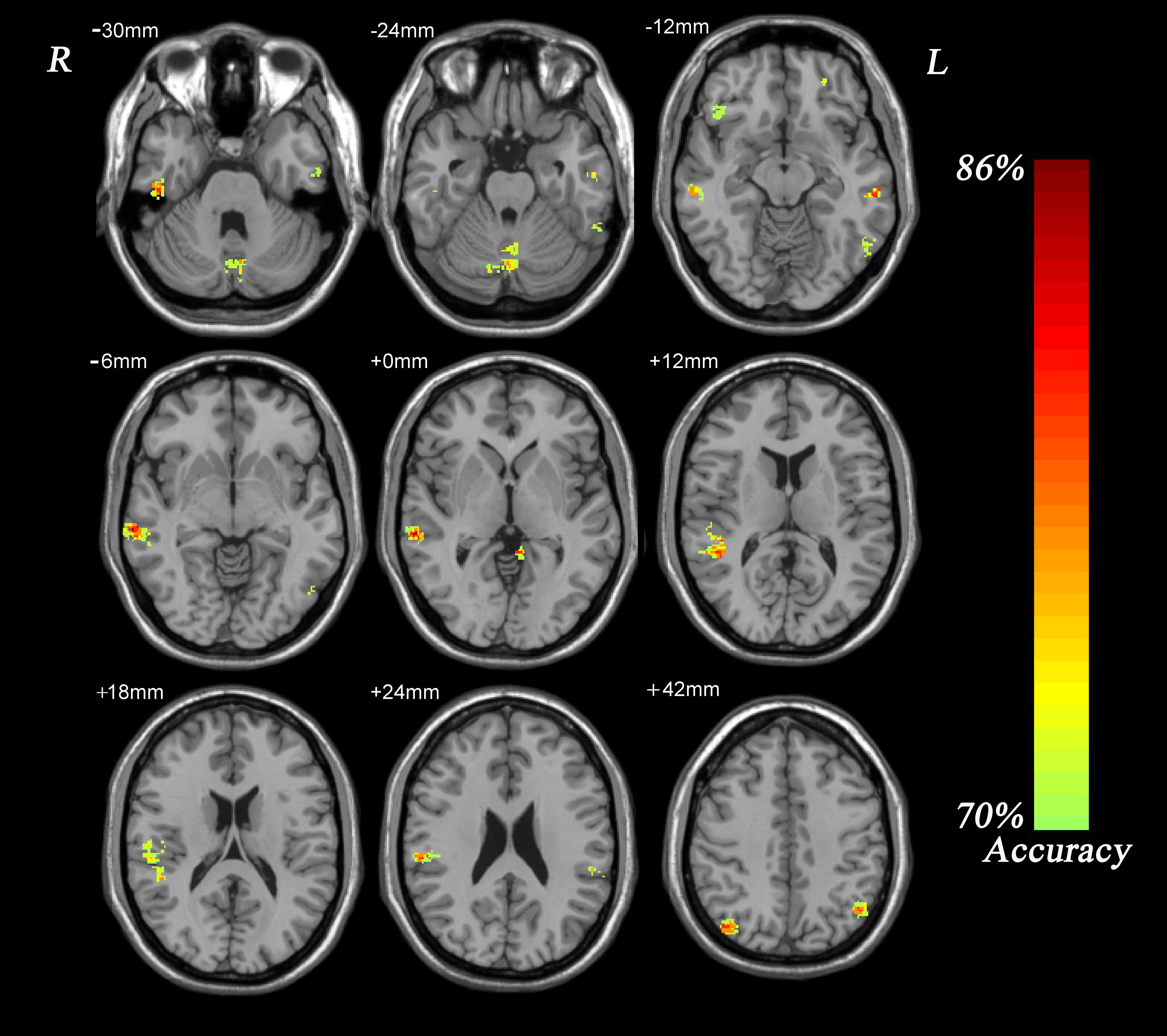

Supplement: Figure S2 — Resulting spatial maps of accuracy for discriminating between TRD patients and healthy controls using gray matter. These clusters were identified by setting the threshold of accuracy higher than 70% and cluster size more than 50 voxels. (TIF) [file pone.0040968.s002.tif]

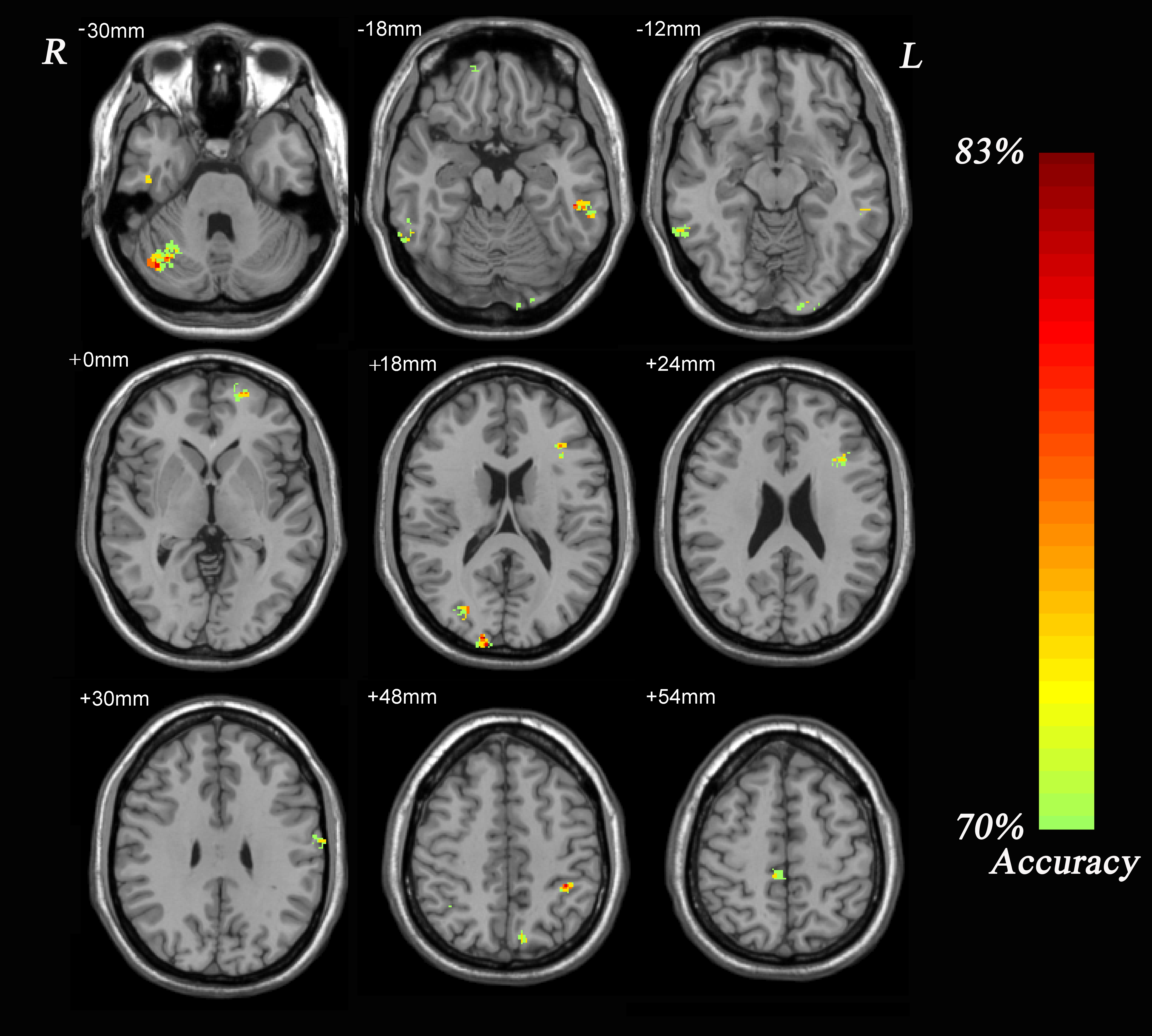

Supplement: Figure S3 — Resulting spatial maps of accuracy for discriminating between TSD patients and healthy controls using gray matter. These clusters were identified by setting the threshold of accuracy higher than 70% and cluster size more than 50 voxels. (TIF) [file pone.0040968.s003.tif]

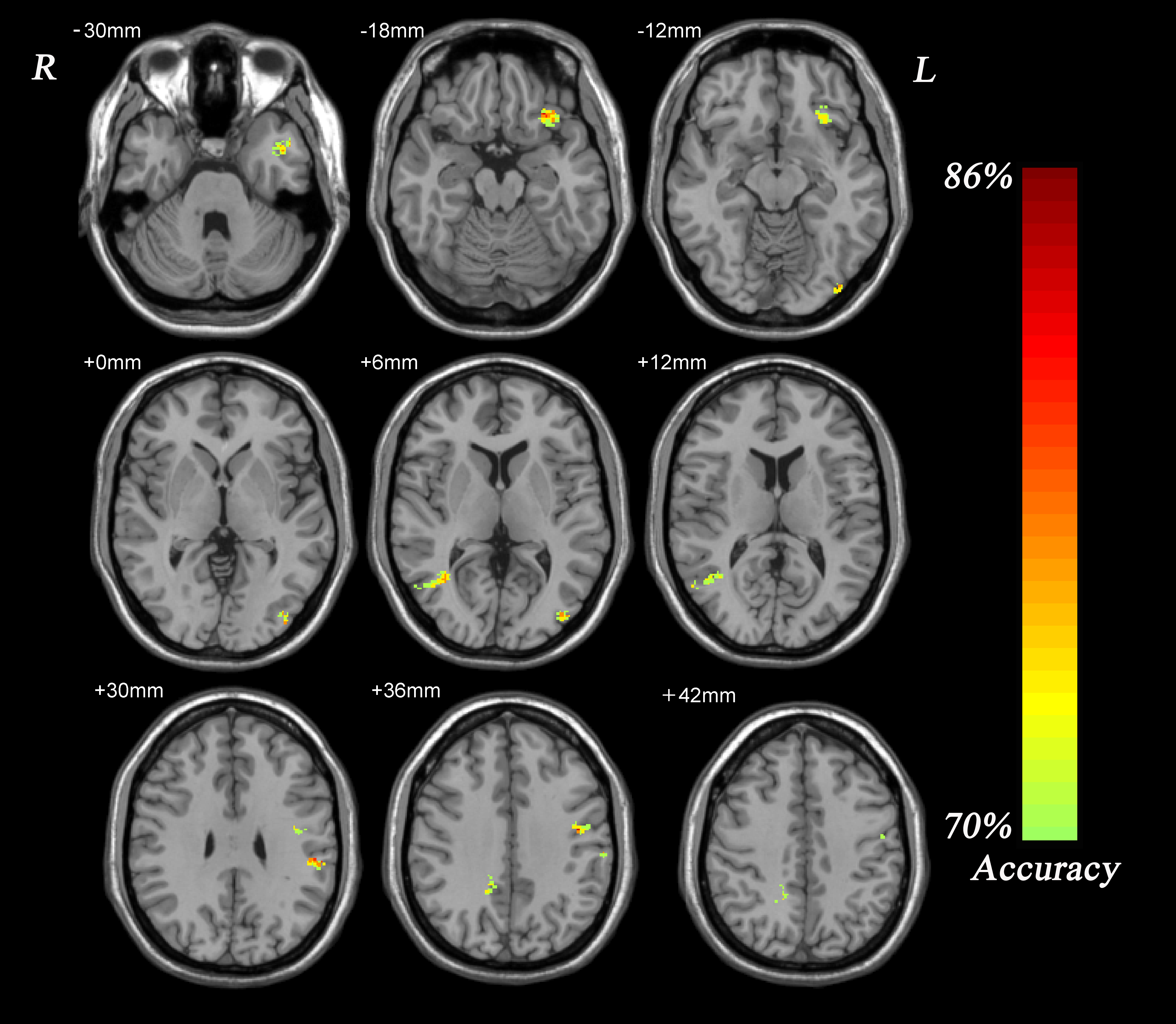

Supplement: Figure S4 — Resulting spatial maps of accuracy for discriminating between TRD patients and healthy controls using white matter. These clusters were identified by setting the threshold of accuracy higher than 70% and cluster size more than 50 voxels. (TIF) [file pone.0040968.s004.tif]

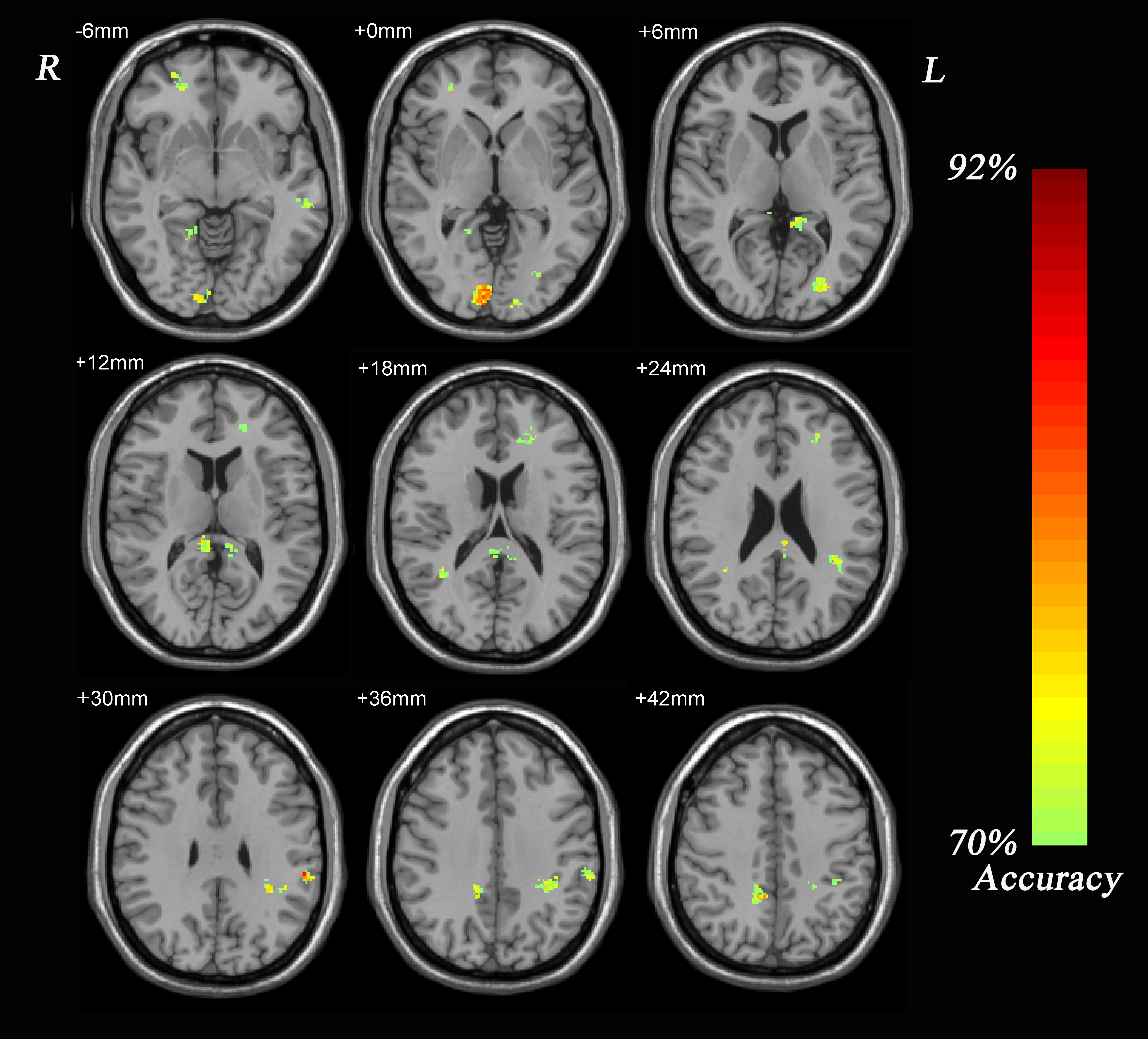

Supplement: Figure S5 — Resulting spatial maps of accuracy for discriminating between TSD patients and healthy controls using white matter. These clusters were identified by setting the threshold of accuracy higher than 70% and cluster size more than 50 voxels. (TIF) [file pone.0040968.s005.tif]
