# Supplementary material for: Classification of Different Therapeutic Responses of Major Depressive Disorder with Multivariate Pattern Analysis Method Based on Structural MR Scans
Source: PLoS One. 2012 Jul 17;7(7):e40968. doi: 10.1371/journal.pone.0040968 (PMC3398877; doi:10.1371/journal.pone.0040968)
Supplement: Table S1 — Most important gray matter regions discriminating between TRD patients and healthy controls. (DOC) [file pone.0040968.s006.doc]

**Table S1.** Most important gray matter regions discriminating between TRD patients and healthy controls.

| Brain regions | BA | Cluster size (voxels) | MNI coordinates (mm) | | | Peak Accuracy(%) | *P* value |
| --- | --- | --- | --- | --- | --- | --- | --- |
|  |  |  | x | y | z |  |  |
| **Frontal** |  |  |  |  |  |  |  |
| Left middle frontal gyrus | 11 | 184 | -25 | 46 | -18 | 82.9 | 0.002 |
| Right inferior frontal gyrus | 47 | 75 | 48 | 22 | -10 | 74.3 | 0.007 |
| **Parietal** |  |  |  |  |  |  |  |
| Left precuneus | 7 | 50 | -12 | -69 | 36 | 77.1 | 0.001 |
| Left supramarginal gyrus | 40 | 119 | -54 | -34 | 24 | 77.1 | 0.001 |
| Right supramarginal gyrus | 40 | 734 | 57 | -27 | 24 | 82.9 | 0.002 |
| Left angular gyrus | 39 | 175 | -46 | -64 | 39 | 80.0 | 0.002 |
| Right angular gyrus | 39 | 414 | 39 | -72 | 37 | 85.7 | 0.001 |
| **Occipital** |  |  |  |  |  |  |  |
| Left lingual gyrus | 17/18 | 59 | -6 | -43 | -1 | 80.0 | 0.001 |
| **Temporal** |  |  |  |  |  |  |  |
| Left middle temporal gyrus | 21 | 73 | -60 | -30 | -12 | 82.9 | 0.001 |
| Right middle temporal gyrus | 21 | 578 | 66 | -28 | -6 | 85.7 | 0.001 |
| Left inferior temporal gyrus | 20 | 371 | -57 | -49 | -22 | 80.0 | 0.002 |
| Right inferior temporal gyrus | 20 | 180 | 51 | -24 | -34 | 82.9 | 0.001 |
| **Cerebellum** |  |  |  |  |  |  |  |
| Left cerebellum posterior lobe | - | 181 | -25 | -58 | -57 | 77.1 | 0.002 |
| Right cerebellum posterior lobe | - | 816 | 18 | -67 | -46 | 80.0 | 0.001 |

The *P* values were obtained by permutation test. BA, Broadmann's area.
