# Supplementary material for: Classification of Different Therapeutic Responses of Major Depressive Disorder with Multivariate Pattern Analysis Method Based on Structural MR Scans
Source: PLoS One. 2012 Jul 17;7(7):e40968. doi: 10.1371/journal.pone.0040968 (PMC3398877; doi:10.1371/journal.pone.0040968)
Supplement: Table S2 — Most important gray matter regions discriminating between TSD patients and healthy controls. (DOC) [file pone.0040968.s007.doc]

**Table S2.** Most important gray matter regions discriminating between TSD patients and healthy controls.

| Brain regions | BA | Cluster size (voxels) | MNI coordinates (mm) | | | Peak Accuracy(%) | *P* value |
| --- | --- | --- | --- | --- | --- | --- | --- |
|  |  |  | x | y | z |  |  |
| **Frontal** |  |  |  |  |  |  |  |
| Left superior frontal gyrus | 8 | 126 | -19 | 60 | 0 | 76.5 | 0.001 |
| Right superior frontal gyrus | 8 | 63 | 13 | 55 | -24 | 76.5 | 0.005 |
| Left inferior frontal gyrus | 47 | 137 | -33 | 20 | 27 | 79.4 | 0.002 |
| Left precentral gyrus | 4/6 | 71 | -63 | -9 | 33 | 76.5 | 0.001 |
| Right paracentral lobule | 5/6 | 68 | 9 | -34 | 55 | 76.5 | 0.001 |
| **Parietal** |  |  |  |  |  |  |  |
| Left angular gyrus | 39 | 85 | -40 | -42 | 49 | 82.4 | 0.005 |
| Right angular gyrus | 39 | 69 | 40 | -52 | 36 | 79.4 | 0.002 |
| Left precuneus | 7 | 85 | -10 | -76 | 46 | 76.5 | 0.001 |
| **Occipital** |  |  |  |  |  |  |  |
| Left lingual gyrus | 17 | 127 | -16 | -97 | -15 | 76.5 | 0.001 |
| Right superior occipital gyrus | 18/19 | 111 | 15 | -96 | 18 | 82.4 | 0.001 |
| Right middle occipital gyrus | 19 | 140 | 32 | -81 | 14 | 76.5 | 0.001 |
| **Temporal** |  |  |  |  |  |  |  |
| Right middle temporal gyrus | 21 | 176 | 55 | -49 | -16 | 79.4 | 0.002 |
| Left inferior temporal gyrus | 20 | 235 | -50 | -33 | -18 | 79.4 | 0.002 |
| Right inferior temporal gyrus | 20 | 52 | 46 | -12 | -33 | 76.5 | 0.001 |
| **Cerebellum** |  |  |  |  |  |  |  |
| Right cerebellum posterior lobe | - | 391 | 42 | -70 | -33 | 79.4 | 0.003 |

The *P* values were obtained by permutation test. BA, Broadmann's area.
