# Supplementary material for: Classification of Different Therapeutic Responses of Major Depressive Disorder with Multivariate Pattern Analysis Method Based on Structural MR Scans
Source: PLoS One. 2012 Jul 17;7(7):e40968. doi: 10.1371/journal.pone.0040968 (PMC3398877; doi:10.1371/journal.pone.0040968)
Supplement: Table S3 — Most important white matter regions discriminating between TRD patients and healthy controls. (DOC) [file pone.0040968.s008.doc]

**Table S3.** Most important white matter regions discriminating between TRD patients and healthy controls.

| Brain regions | BA | Cluster size (voxels) | MNI coordinates (mm) | | | Peak Accuracy(%) | *P* value |
| --- | --- | --- | --- | --- | --- | --- | --- |
|  |  |  | x | y | z |  |  |
| **Frontal** |  |  |  |  |  |  |  |
| Left inferior frontal gyrus | 47 | 255 | -28 | 27 | -18 | 82.9 | 0.001 |
| Left precentral gyrus | 6 | 142 | -42 | -9 | 31 | 82.9 | 0.002 |
| **Parietal** |  |  |  |  |  |  |  |
| Left postcentral gyrus | 5/7 | 348 | -15 | -43 | 69 | 85.7 | 0.001 |
| Left supramarginal gyrus | 40 | 92 | -55 | -31 | 31 | 82.9 | 0.001 |
| Right posterior cingulate gyrus | 31 | 67 | 10 | -46 | 37 | 77.1 | 0.001 |
| **Occipital** |  |  |  |  |  |  |  |
| Left middle occipital gyrus | 19 | 117 | -42 | -84 | 3 | 80.0 | 0.001 |
| Left inferior occipital gyrus | 18/19 | 107 | -39 | -87 | -9 | 82.9 | 0.001 |
| **Temporal** |  |  |  |  |  |  |  |
| Left middle temporal gyrus | 21 | 184 | -40 | 4 | -33 | 82.9 | 0.001 |
| Right middle temporal gyrus | 21/22 | 258 | 39 | -60 | 4 | 77.1 | 0.001 |
| **Cerebellum** |  |  |  |  |  |  |  |
| Left cerebellum posterior lobe | - | 252 | -12 | -67 | -55 | 82.9 | 0.001 |
| Right cerebellum posterior lobe | - | 122 | 33 | -70 | -52 | 74.3 | 0.004 |
| Pons | - | 148 | 10 | -24 | -40 | 77.1 | 0.001 |

The *P* values were obtained by permutation test. BA, Broadmann's area.
