# Supplementary material for: Classification of Different Therapeutic Responses of Major Depressive Disorder with Multivariate Pattern Analysis Method Based on Structural MR Scans
Source: PLoS One. 2012 Jul 17;7(7):e40968. doi: 10.1371/journal.pone.0040968 (PMC3398877; doi:10.1371/journal.pone.0040968)
Supplement: Table S4 — Most important white matter regions discriminating between TSD patients and healthy controls. (DOC) [file pone.0040968.s009.doc]

**Table S4.** Most important white matter regions discriminating between TSD patients and healthy controls.

| Brain regions | BA | Cluster size (voxels) | MNI coordinates (mm) | | | Peak Accuracy(%) | *P* value |
| --- | --- | --- | --- | --- | --- | --- | --- |
|  |  |  | x | y | z |  |  |
| **Frontal** |  |  |  |  |  |  |  |
| Right rectal gyrus | 11 | 76 | 6 | 40 | -27 | 76.5 | 0.001 |
| Right supplementary motor area | 6 | 57 | 9 | 3 | 58 | 76.5 | 0.001 |
| Left anterior cingulate gyrus | 32 | 165 | -27 | 39 | 21 | 79.4 | 0.002 |
| Right middle frontal gyrus | 11 | 73 | 27 | 46 | -7 | 73.5 | 0.001 |
| **Parietal** |  |  |  |  |  |  |  |
| Left precuneus | 7 | 108 | -9 | -42 | 4 | 76.5 | 0.001 |
| Right precuneus | 7/31 | 256 | 10 | -48 | 39 | 82.4 | 0.001 |
| Left inferior parietal lobule | 40 | 350 | -29 | -39 | 33 | 79.4 | 0.001 |
| Left supramarginal gyrus | 40 | 169 | -57 | -31 | 31 | 91.2 | 0.001 |
| Right posterior cingulate gyrus | 23/31 | 138 | 6 | -39 | 10 | 76.5 | 0.001 |
| **Occipital** |  |  |  |  |  |  |  |
| Left lingual gyrus | 17/18 | 128 | -9 | -88 | -18 | 76.5 | 0.001 |
| Right lingual gyrus | 19 | 92 | 19 | -51 | -10 | 76.5 | 0.001 |
| Right calcarine fissure | 17 | 326 | 7 | -87 | 1 | 85.3 | 0.001 |
| Left middle occipital gyrus | 19 | 131 | -31 | -82 | 3 | 79.4 | 0.001 |
| **Temporal** |  |  |  |  |  |  |  |
| Right superior temporal gyrus | 22 | 65 | 36 | -49 | 22 | 76.5 | 0.001 |
| Left middle temporal gyrus | 21 | 107 | -58 | -31 | -9 | 79.4 | 0.001 |

The *P* values were obtained by permutation test. BA, Broadmann's area.
