# Supplementary material for: Classification of Different Therapeutic Responses of Major Depressive Disorder with Multivariate Pattern Analysis Method Based on Structural MR Scans
Source: PLoS One. 2012 Jul 17;7(7):e40968. doi: 10.1371/journal.pone.0040968 (PMC3398877; doi:10.1371/journal.pone.0040968)
Supplement: Table S5 — Brain regions showing gray matter volume differences in TRD patients compared with TSD patients. (DOC) [file pone.0040968.s010.doc]

**Table S5.** Brain regions showing gray matter volume differences in TRD patients compared with TSD patients.

| Brain regions | BA | Cluster size (voxels) | MNI coordinates (mm) | | | T value |
| --- | --- | --- | --- | --- | --- | --- |
|  |  |  | x | y | z |  |
| **Frontal** |  |  |  |  |  |  |
| Left middle frontal gyrus | 9 | 7 | -33 | 21 | 35 | 3.58 |
| **Parietal** |  |  |  |  |  |  |
| Right supramarginal gyrus | 40 | 8 | 45 | -34 | 42 | -3.66 |
| Right inferior parietal lobule | 39/40 | 13 | 39 | -37 | 49 | -3.71 |
| **Occipital** |  |  |  |  |  |  |
| Left superior occipital gyrus | 18 | 25 | -10 | -108 | 10 | 3.78 |
| Left calcarine fissure | 17 | 15 | -10 | -99 | -9 | 3.51 |
| **Temporal** |  |  |  |  |  |  |
| Left middle temporal gyrus | 21 | 38 | -66 | -50 | -6 | -3.97 |
| Left inferior temporal gyrus | 20 | 10 | -58 | -6 | -28 | -3.55 |
| **Subcortical** |  |  |  |  |  |  |
| Right caudate nucleus | - | 89 | 6 | 6 | 0 | -4.22 |

T statistical value of peak voxel showing gray matter volume differences between the TRD patients and TSD. TRD, treatment-resistant depression; TSD, treatment-sensitive depression; BA, Broadmann's area. *p*<.001, uncorrected. Of note, we showed the two-sample *t*-tests results within the identified gray matter regions by using MVPA between TRD and TSD patients (see Table 2).
