# Supplementary material for: Classification of Different Therapeutic Responses of Major Depressive Disorder with Multivariate Pattern Analysis Method Based on Structural MR Scans
Source: PLoS One. 2012 Jul 17;7(7):e40968. doi: 10.1371/journal.pone.0040968 (PMC3398877; doi:10.1371/journal.pone.0040968)
Supplement: Table S6 — Brain regions showing white matter volume differences between the TRD patients and TSD patients. (DOC) [file pone.0040968.s011.doc]

**Table S6.** Brain regions showing white matter volume differences between the TRD patients and TSD patients.

| Brain regions | BA | Cluster size (voxels) | MNI coordinates (mm) | | | T value |
| --- | --- | --- | --- | --- | --- | --- |
|  |  |  | x | y | z |  |
| **Frontal** |  |  |  |  |  |  |
| Right medial frontal gyrus | 11 | 9 | 10 | 34 | -19 | 3.45 |
| Right middle frontal gyrus | 9 | 11 | 36 | 45 | 1 | 3.49 |
| Right middle frontal gyrus | 8 | 60 | 27 | 28 | 46 | 4.81 |
| Left anterior cingulate gyrus | 32 | 43 | -21 | 30 | 25 | 3.70 |
| Left median cingulate gyrus | 24 | 13 | -10 | -18 | 40 | 3.73 |
| Right median cingulate gyrus | 24 | 64 | 7 | 1 | 42 | 4.90 |
| Left precentral gyrus | 6 | 11 | -39 | -6 | 49 | 4.28 |
| **Parietal** |  |  |  |  |  |  |
| Left supramarginal gyrus | 40 | 22 | -39 | -52 | 22 | 3.84 |
| Left precuneus | 7 | 19 | -19 | -70 | 49 | 3.95 |
| Left posterior cingulate gyrus | 23/31 | 70 | -4 | -36 | 24 | 3.61 |
| **Occipital** |  |  |  |  |  |  |
| Left lingual gyrus | 17/18 | 220 | -12 | -91 | -21 | 5.07 |
| Right lingual gyrus | 17 | 25 | 9 | -93 | 0 | -3.92 |
| Left middle occipital gyrus | 18 | 17 | -39 | -88 | -3 | 3.98 |

T statistical value of peak voxel showing gray matter volume differences between the TRD patients and TSD. TRD, treatment-resistant depression; TSD, treatment-response depression; BA, Broadmann's area. *p*<.001, uncorrected. Of note, we showed the two-sample *t*-tests results within the identified white matter regions by using MVPA between TRD and TSD patients (see Table 3).
