# Supplementary material for: Classification of Different Therapeutic Responses of Major Depressive Disorder with Multivariate Pattern Analysis Method Based on Structural MR Scans
Source: PLoS One. 2012 Jul 17;7(7):e40968. doi: 10.1371/journal.pone.0040968 (PMC3398877; doi:10.1371/journal.pone.0040968)
Supplement: Table S7 — Correlation between HRSD scores and gray matter volume in TRD and TSD patients. (DOC) [file pone.0040968.s012.doc]

**Table S7.** Correlation between HRSD scores and gray matter volume in TRD and TSD patients.

| Brain regions | BA | Cluster size (voxels) | MNI coordinates (mm) | | | T value |
| --- | --- | --- | --- | --- | --- | --- |
|  |  |  | x | y | z |  |
| Positive correlation between HRSD scores and gray matter volume in TRD and TSD patients | | | | | | |
| Left cerebellum posterior lobe | - | 28 | -18 | -51 | -60 | 2.39 |
| Right cerebellum posterior lobe | - | 13 | 9 | -57 | -55.5 | 2.41 |
| Left inferior frontal gyrus | 47 | 114 | -43.5 | 25.5 | -9 | 2.85 |
| Right superior temporal gyrus | 13/41 | 21 | 49.5 | -37.5 | 21 | 4.19 |
| Left inferior parietal lobule | 40 | 18 | 54 | -46.5 | 24 | 3.95 |

HRSD, Hamilton Rating Scale for Depression; TRD, treatment-resistant depression; TSD, treatment-sensitive depression. *p*<.05, Alphasim corrected. Of note, we implemented the correlation analysis within the identified gray matter regions by using MVPA between TRD and TSD patients (see Table 2).
