# Supplementary material for: Classification of Different Therapeutic Responses of Major Depressive Disorder with Multivariate Pattern Analysis Method Based on Structural MR Scans
Source: PLoS One. 2012 Jul 17;7(7):e40968. doi: 10.1371/journal.pone.0040968 (PMC3398877; doi:10.1371/journal.pone.0040968)
Supplement: Table S8 — Correlation between HRSD scores and white matter volume in TRD and TSD patients. (DOC) [file pone.0040968.s013.doc]

**Table S8.** Correlation between HRSD scores and white matter volume in TRD and TSD patients.

| Brain regions | BA | Cluster size (voxels) | MNI coordinates (mm) | | | T value |
| --- | --- | --- | --- | --- | --- | --- |
|  |  |  | x | y | z |  |
| Positive correlation between HRSD scores and gray matter volume in TRD and TSD patients | | | | | | |
| Right cuenus | 17 | 10 | 7.5 | -96 | 0 | 2.45 |
| Negative correlation between HRSD scores and gray matter volume in TRD and TSD patients | | | | | | |
| Left medial frontal gyrus | 32 | 239 | -22 | 39 | 9 | -2.81 |
| Left median cingulate gyrus | 24 | 13 | -15 | -19 | 40 | -2.65 |

HRSD, Hamilton Rating Scale for Depression; TRD, treatment-resistant depression; TSD, treatment-sensitive depression. *p*<.05, Alphasim corrected. Of note, we implemented the correlation analysis within the identified white matter regions by using MVPA between TRD and TSD patients (see Table 3).
